# A Just-in-Time Adaptive Intervention (Shift) to Manage Problem Anger After Trauma: Co-Design and Development Study

**DOI:** 10.2196/62960

**Published:** 2025-05-22

**Authors:** Olivia Metcalf, David Forbes, Lauren M Henry, Tianchen Qian, Tracey Varker, Melissa A Brotman, Sean Cowlishaw, Karen E Lamb, Meaghan L O'Donnell

**Affiliations:** 1 Centre for Digital Transformation of Health, University of Melbourne, 700 Swanston St, Carlton, 3053, Australia, 61 404040817; 2 Phoenix Australia - Centre for Posttraumatic Mental Health, University of Melbourne, Carlton, Australia; 3 Neuroscience and Novel Therapeutics Unit, Emotion and Development Branch, National Institute of Mental Health Intramural Research Program, Bethesda, MD, United States; 4 Department of Statistics, University of California, Irvine, Irvine, CA, United States; 5 Monash School of Psychological Sciences, Turner Institute for Brain and Mental Health, Monash University, Clayton, Australia; 6 MISCH (Methods and Implementation Support for Clinical Health) Research Hub, Faculty of Medicine, Dentistry and Health Sciences, University of Melbourne, Carlton, Australia

**Keywords:** co-design, just-in-time adaptive intervention, mental health, anger, trauma, design, tool, digital tool, post-traumatic, mental health, participatory design, development, user, support, feedback, mood, monitoring, manage

## Abstract

**Background:**

Problem anger is common after experiencing trauma and is under-recognized relative to other posttraumatic mental health issues. Previous research has shown that digital mental health tools have significant potential to support individuals with problem anger after trauma.

**Objective:**

The objective of this study was to describe the co-design and development of a just-in-time adaptive intervention (JITAI) targeting problem anger in individuals who have experienced trauma.

**Methods:**

We used a participatory design process following the double-diamond framework. Phase 1 involved one-on-one qualitative interviews with trauma-exposed individuals with problem anger (n=10). Using an inductive approach (interpretative phenomenological analysis), we thematically coded interview data to create design principles for this population and generate potential content for the intervention. Phase 2 involved academic and clinical experts in trauma and experts in digital health reviewing the Phase 1 results and an evidence-based cognitive behavioral approach to treating anger. We then created intervention content and prototypes, which we then took to workshops with all participants for feedback, using group discussions and ratings of desirability and feasibility.

**Results:**

From Phase 1, core considerations for a JITAI included look and feel preferences, self-led and personalized support and content, and different support needed for each anger stage. A JITAI was developed with the following components: (1) personalized schedules and content onboarding; (2) psychoeducation about problem anger; (3) crisis support; (4) mood monitoring via anger check-ins; (5) self-led and personalized circuit breakers; (6) cognitive-behavioral based skills; (7) and a digital Coach embedded in the app. Some suggested features, such as social networking and sharing data with loved ones, were not pursued due to feasibility reasons relating to participant safety or technical costs.

**Conclusions:**

The resulting JITAI, termed “Shift*,”* is the first digital mental health tool designed with end users to manage anger after trauma.

## Introduction

Problem anger, defined as anger occurring at an intensity, frequency, and duration that negatively impacts an individual and their relationships, is one of the most common posttraumatic mental health issues, and is highly prevalent in predominately male military and first responder populations [1,2], as well as women who have experienced trauma [3]. While cognitive behavioral therapy (CBT)-based treatments can be effective, there are difficulties engaging individuals with problem anger in treatment due to impacts of hostility, mistrust, and interpersonal conflicts on therapeutic relationships [4]. Mental health practitioners commonly report feeling more drained, more therapeutic ruptures, and providing shorter treatments in response to antagonistic client presentations, resulting in worse outcomes [5].

Advancements in digital mental health tools may provide nontraditional treatment options for problem anger [6]. As an emotion, anger is unique because it manifests as primarily externally-focused, in that an external agent often activates anger, such as how other people have behaved in a hurtful, disrespectful, or otherwise anger-triggering way [7]. Accordingly, interventions that increase internal focus are likely to support greater self-awareness to offset focus on anger-provoking events. Early efforts in this area used digital technologies such as smartphones and wearables to record physiological data, monitor symptoms, and support practicing of CBT-based skills as an adjunct to traditional CBT, and while effective in reducing anger symptoms, did not have additive effects beyond standard psychological treatment [8]. Recent research using wearables and regular mood monitoring delivered via smartphone have shown promise [9]. Despite the potential, there are many challenges to the development of innovative digital mental health tools for anger. First, the overwhelming majority of research into digital mental health tools has focused on depression and anxiety, and the foundation for building anger-focused digital solutions is limited and nonspecific [10]. Second, digital mental health tools more broadly face significant challenges around uptake and usage [11]. Modest engagement with digital mental health apps is due to a variety of reasons including that may include a lack of user involvement in the design, as well as lack of tailoring to suit the times an individual most needs support [12]. As such, digital mental health tools that are designed alongside users and support real-world moments of high need may be beneficial.

JITAI are 1 type of new digital health approach that may overcome challenges associated with low engagement by providing the right information at the right time [13]. JITAIs deliver intervention content that are adapted to an individual’s context, such as the level and type of support matched to an individual’s current emotional state [14]. Efforts to develop such interventions in physical activity [15], depression [16], substance use [17], and adolescent mental health [18] have shown promise in improving affect, cognition, and behavior. A JITAI includes 6 key elements, namely a distal outcome, proximal outcomes, decision points, intervention options, tailoring variables, and decision rules. While research has established that digital mental health tools for problem anger are feasible, much remains unknown about how to design a JITAI for this population, and more empirical work including data-driven approaches to designing JITAI is needed [14,17]. The aim of this study was to understand the tailoring variables and co-design the intervention options of a JITAI with individuals who have experienced trauma and problem anger, alongside experts in digital and mental health, by understanding:

1. What is the preferred language, visual features, and content of a digital mental health tool to help individuals manage their problem anger?
2. When do individuals need support to manage their anger differently?
3. Taking the findings from above, and combining them with evidence-based CBT-approaches, what is acceptable and feasible content for a JITAI for problem anger?

## Methods

### Overview

This study had 2 phases, following the double diamond framework (ie, discover, define, develop, and deliver) [19] which is user-centered and well validated in digital health tool research [20]. Phase 1 involved qualitative interviews with individuals with problem anger after trauma (termed “experts by experience”) to understand their experiences with digital mental health tools and treatment for anger, their “pain points” regarding day-to-day management of their anger, and views about essential content, features, and functionality components of smartphone-delivered interventions for problem anger. Phase 2 involved co-design workshops with a mixture of experts by experience, and experts by profession, which included digital health experts, trauma researchers, and clinical psychologists to converge on core components of a prototype JITAI, using CBT for anger after trauma principles [21]. Both phases were conducted in line with trauma-informed research principles outlined in the recent Trauma and Resilience Informed Research Principles and Practice (TRIRPP) Framework [22], which aims to improve participation in research by individuals who have experienced trauma and to avoid retraumatization of participants. Specifically, our study involved actively minimizing retraumatization by structuring interview guides so that participants do not have to discuss their trauma, framing the research process as relational, addressing power imbalances by having twice the number of experts by experience than experts by profession, and making available women-identifying groups only for the group sessions, recognizing many women experience trauma as a result of gendered violence. We also made available individual co-design workshops for participants who did not want to engage in group work for any reason.

### Phase 1

#### Participants

A total of 10 experts by experience (5 out of 10 were women; aged 19‐49 years old) with a history of trauma exposure and problem anger were invited to participate via the researchers’ database, which contained a list of individuals who had participated in previous trauma and anger–related research at the University of Melbourne. Participants needed to meet the criteria for problem anger and have reported experiencing a traumatic event previously, be residing in Australia, have access to Zoom, be over the age of 18 years, and be fluent in English. All participants had sought help for their mental health previously, and 4 had received a diagnosis of posttraumatic stress disorder. One individual identified as living with a disability. In addition, 8 participants had previously tried apps to support their mental health. All participants verbally consented to participate in the qualitative interviews. Data were collected virtually via Zoom (Zoom Communications) using audio-video recordings across August-September 2023 by author OM using a semistructured interview guide and interviews lasted approximately 45 minutes. Interview guides were developed by the research team after reviewing the literature around problem anger experiences, and digital mental health tools. Interview guides are in Multimedia Appendix 1.

#### Analyses

Qualitative interview data were transcribed verbatim from audio-video recordings. Data were analyzed using NVivo (Lumivero) and using an inductive approach (interpretive phenomenological analysis) by 2 independent coders who were part of the research team, to allow identification of themes about the experiences of problem anger treatments, and views and preferences about content, features, and functionality of the JITAI [23]. Each coder read the transcripts, coded the insights, and clustered the codes into themes. Coders discussed consensus at the insights and themes stage to reach convergence. No formal inter-rater reliability was conducted.

### Phase 2

#### Participants

All participants from Phase 1 were invited to attend Phase 2 and agreed to participate. In addition, 6 experts by profession (ie, clinicians and researchers in problem anger and digital health) were invited to participate. This number was determined to balance the power dynamics between expert types and to ensure experts by experience were not outnumbered in workshops. Experts by profession were identified by the research team based on their expertise in the field of anger, trauma, or digital health and invited directly. Data were collected virtually across October 2023. Focus group sizes varied between 4‐6 individuals per group and lasted approximately 90 minutes and were conducted via Zoom. Phase 2 were not audio-recorded.

#### Analyses

The themes from Phase 1 were reviewed by the research team alongside the content from an evidence-based CBT manual developed by one of the authors (DF) for managing anger after trauma [21] to distill into a range of possible content features that could help users manage their anger. These content features, as well as 3 different aesthetics styles developed by the digital health experts after reviewing the Phase 1 data, were then taken to Phase 2 workshops in the form of low fidelity prototypes for discussion about content to keep, alter, or remove [24], with the goal of converging on a final prototype. The app title, and the stages of anger was also the focus of the workshops, in terms of converging on a consensus for how to represent them. Finally, the workshop participants charted components of the possible prototype on a 2 by 2 feasibility (low to high) and desirability (low to high) matrix. Workshop discussions were captured as detailed written notes by the facilitator [OM] and another member of the research team who was observing, and comments typed into the Zoom chat function from participants within each of the workshops relating to suggestions to keep, alter, or remove aspects of content, and the charting results of feasibility and acceptability.

### Ethical Considerations

This study was approved by the University of Melbourne Human Research Ethics Committee (Approval number: 2023-26108-39829-4). Written informed consent was obtained for the study. Experts by experience were reimbursed for their time in the form of a voucher ($96.06; AUD $1 = US $1.56) for Phase 1 and 2, while experts by profession were not.

## Results

### Phase 1

The results from the individual interviews revealed several themes, listed in Table 1, relating to preferences for a JITAI for anger. First, participants described wanting an app that was free, with no ads, and easy to use. Second, several themes relating to design principles were identified. These included the “Look & Feel,” which detailed specific preferences for the language and imagery used in the app. Experts by experience felt that many mental health apps overused the word “calm” and imagery with strong meditation and wellness themes, as well as being too directive, using terms such as “should” and “need” to direct users. As a result, they felt that many mental health apps were condescending and irritating, and worsened anger when used for self-management. The theme “Strengths-based” described preferences of experts by experience for an app that felt supportive and addressed the guilt and shame they felt about their anger. Experts by experience reflected wanting digital mental health tools that feel like a companion: “a trusted mate.” Under the theme of “Personalized*,”* there was divergence regarding the types of content individuals wanted, such as military and first-responder-specific information explaining the role of occupation in anger, the links between anger and substance use, as well as menstrual health. Strongly linked was the theme “Self-led” which encompassed accounts of who support should come from and how information should be delivered, particularly during heightened states of anger. While the voice of professional experts and lived experience was valued, overwhelmingly, experts by experience conveyed that the person they would listen to was themselves. Conversely, some experts by experience conveyed how complex trauma left them uncertain about trusting their own voice, and thus they would want information delivered to them by an avatar they could create.

**Table 1.** Design principles and desired intervention components from qualitative interviews with ten participants aged 19‐49 years old with a history of trauma and problem anger.

Themes	Example quote
Design principles	
Free and no ads	—^a^
Easy to use	*The most annoying and triggering thing about using an app is when it doesn’t do what you want it to do, or you’re trying to do something, and then you[‘re] hit with roadblocks*. [Expert by Experience 1]
Look & Feel	*It would have to be written in a way that it comes from someone that isn’t condescending; that doesn’t make you angry reading it*. [Expert by Experience 1]*It tells you to take three deep breaths when you log in on that [mental health app]. I’m like, I don’t want to take three deep breaths. I can’t. When I breathe, it makes me angry*. [Expert by Experience 2]
Strengths-based	*I’m not going to use anything that is just focused on my anger. Like who would want that, just some negative app going [beep] ‘you are mad again’. [laughs] praise me for stuff too*. [Expert by Experience 2]
Personalized	*One option could be you could tick in the app, ‘these are the things that really affect me and are related to my anger.’ And for you, that might be anxiety, it might not be pain or sleep. And then you could also perhaps get support specifically around that. So you have a really personalized kind of list*. [Expert by Experience 1]
Self-led	*Don’t tell me what to do straight up*. [Expert by Experience 2]*I don’t want to listen to some expert, no offence. I don’t read your textbooks, I don’t like studies, theses. I didn’t read them*. [Expert by Experience 7]
Preferred features of an anger-focused app	
Anger is experienced in distinct stages with different support needed for each stage	*‘1 to 10’ for my anger- that doesn’t mean anything to me. It’s out of control, or it’s not*. [Expert by Experience 3]*I want [an app] that … would recognize over time –‘okay, he needs an aid, so I sort of guess he’s feeling these sorts of things’. And then it would give me something structured more for that for that level for me, rather than just a generic one*. [Expert by Experience 4]
Targeting cognition and behavioral aspects of anger	*Rumination is something I’ve had to work really hard on, because it’s caused me a lot of problems recently…I like to say to myself, ‘it’s done. It’s finished, you’re safe, it’s past.’ And I almost take it out of myself and then put it down and say I’m leaving it there. And it’s not going any further. And then when I catch myself ruminating about it if I get home, I say no, that’s outside of I haven’t brought that in I’m not gonna let that come into my calm area of home*. [Expert by Experience 3]
Leveraging physiological data	*I’ve used a wearable and with the blood pressure and seeing the heartrate, that’s very intriguing*. [Expert by Experience 6]
Complimenting current mental health treatments	*If I trusted [a psychologist] and felt like things were progressing, [an app] would be something that I would seek out her opinion on*. [Expert by Experience 7]
Regular check ins	*The process of a check-in and reflecting your anger [is] good, because I never really do that. Like, I don’t think anyone does. I don’t think anyone really sits there and reflects on how they’re feeling or anything like that*. [Expert by Experience 5]
Add-ons	
Social features with other users	*An app that could connect us all, so we don’t feel like we’re all alone, that we still got an identity, something that although we’re doing the app by ourselves, but gives us a feeling that we’re not by ourselves*. [Expert by Experience 7]
Sharing data with loved ones	*I would like the app to send something to my husband saying I’ve had a shocking day, because then that way, he’ll be prepared before he comes home*. [Expert by Experience 3]
Parenting support	*This is hard for me to admit, but my anger with my kids is such a huge trigger [pause interview due to distress]. Yeah, so anyway, things that help me be a better mum*. [Expert by Experience 8]

a —: not available.

In addition to these design features, several other themes emerged in relation to a JITAI for anger. In identifying opportunities to intervene, participants reported that their anger is experienced in distinct stages, highlighting that for them, anger is either increasing, or completely out of control, with different support needed for each stage. Participants also wanted support in the form of ”targeting cognition and behavioral aspects of anger” such as rumination and supporting interactions with others when angry, ”leveraging physiological data” to detect their mood, an app that was “complimenting current mental health treatments,” and “regular check ins” of their anger.

### Phase 2

**Figure 1.**
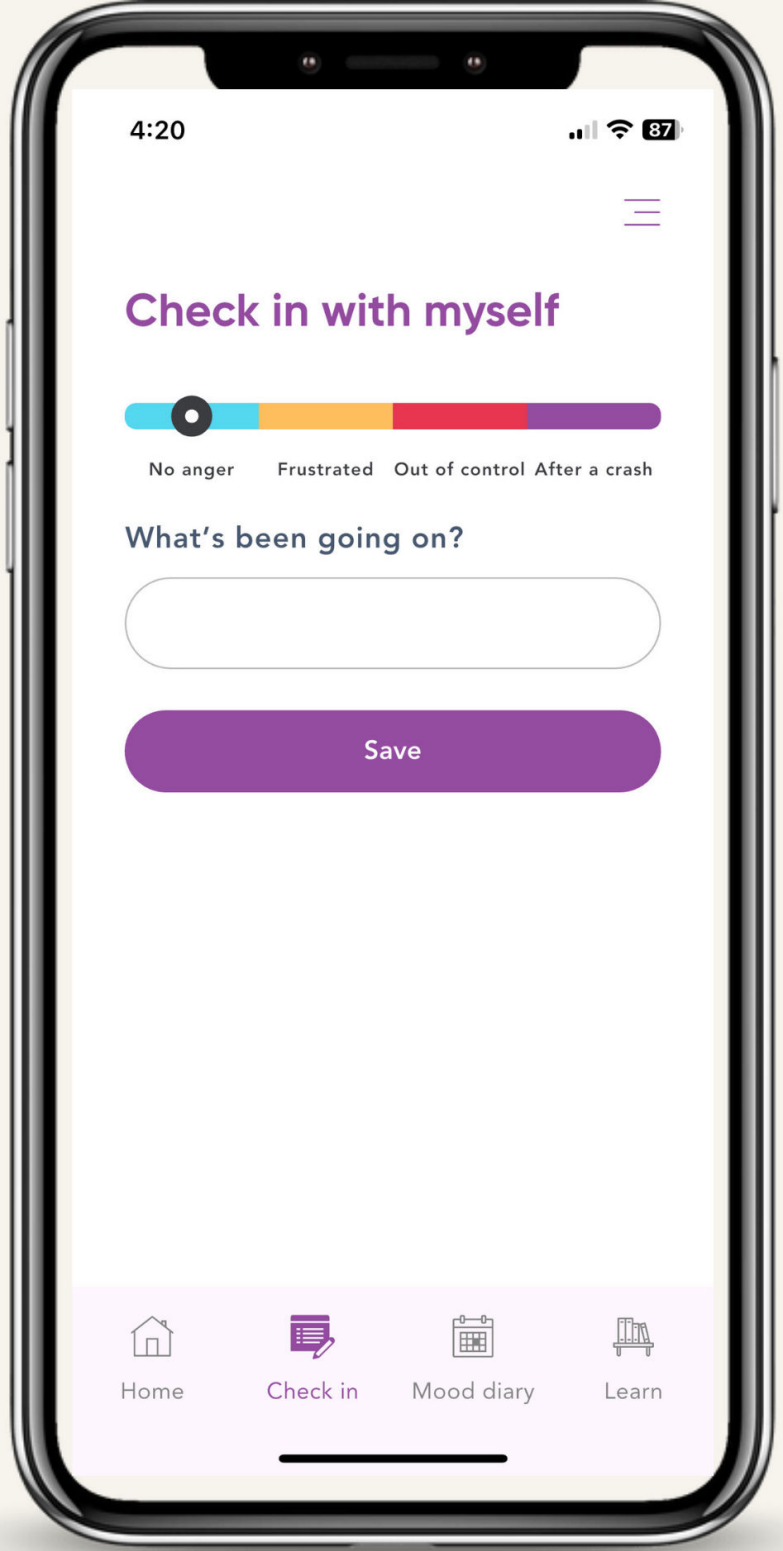
The tailoring variables of the “Shift” just-in-time adaptive intervention in the form of anger stages.

After reviewing Phase 1 results, the trauma experts selected content from an anger-focused CBT-based manual [21] that related to psychoeducation, managing rumination, circuit breakers, cognitive reframing, managing physiological arousal, and assertive communication. This potential content and low-fidelity prototype design options were taken to co-design workshops. The results from the co-design workshop included convergence on the way anger stages should be represented, convergence on the look and feel of the app, convergence on the CBT-based content, and discussions around the feasibility of add-ons. While experts by profession discussed anger in terms of Likert scales (ie, 1 to 10), the experts by experience spoke about how “anger was experienced in stages*,”* with some individuals likening this to a runaway train. The workshops converged on the following stages of anger, shown in Figure 1: (1) “no anger,” (2) “frustrated” (ie, anger is still within an individual’s control, and while heightened, the individual can engage in cognitive, behavioral, or physiological activities), (3) “out of control” (ie, the individual and/or others are not safe), and (iv) “after a crash” (ie, anger at others has dissipated, and feelings of shame, regret, guilt, anger at self, sadness, and loneliness are high). In terms of content, all the recommended CBT skills were considered feasible. While the add-ons such as social networking and sharing data with loved ones was highly desirable by experts by experience, they were rated low in feasibility by the experts by profession, due to technical challenges, cost, and the lack of information on how to protect users’ safety, so were not included. Parenting support was considered as out of scope for the first iteration of the app.

**Figure 2.**
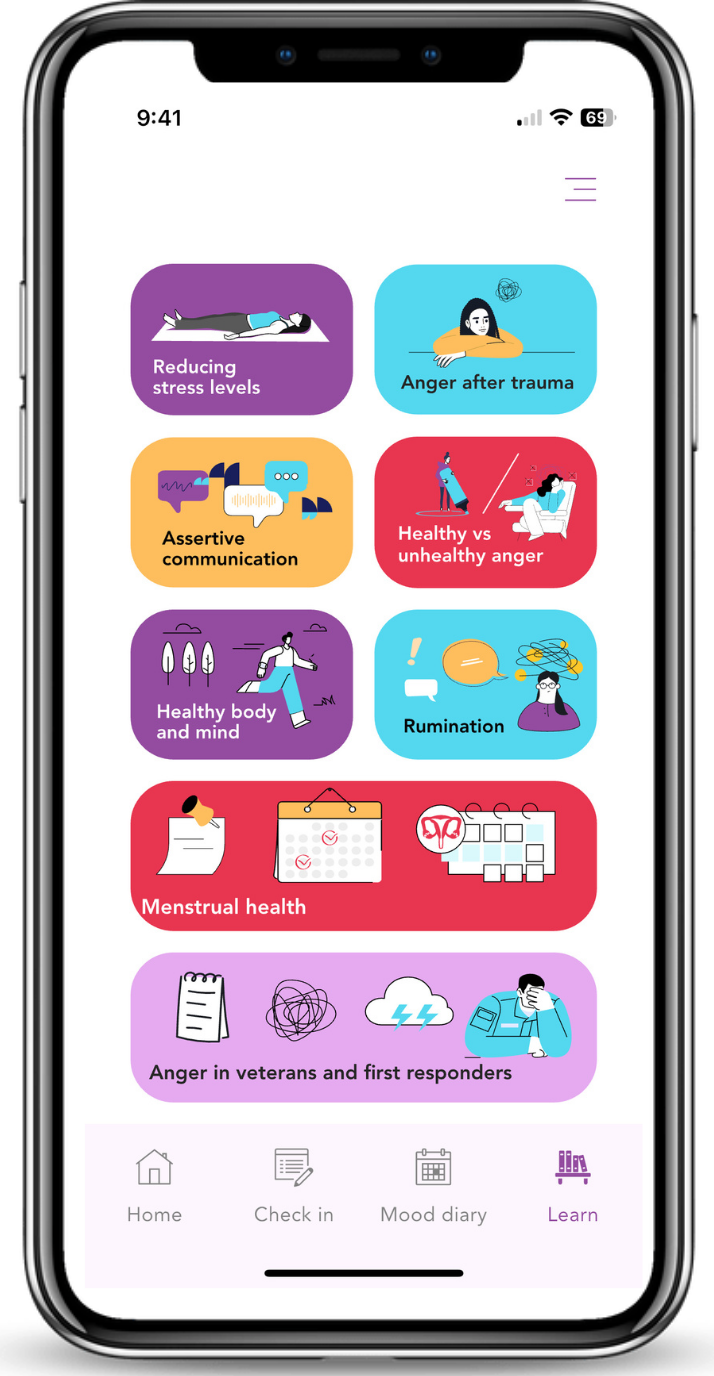
Home screen and psychoeducation section of “Shift.”

The results of both phases led to the development of a JITAI named “Shift” (Figure 2 and Textbox 1)*,* combines mood monitoring (ie, 4 daily check-ins on anger stages, with ability to check-in outside of these 4) with tailored CBT-based support based on the stage of anger. When users check-in as “frustrated,” 6 CBT and arousal management skills, under the categories of “Body, Mind, and Actions” in (Textbox 1) are made available. When users check-in as “out of control,” a personalized circuit breaker preset by the user at the download of the app sends a safe, supportive message, video, photo, image, or song to circuit break the situation. And when users check-in as “after a crash,” warm and emphatic audio messages from their “Shift” Coach are sent reassuring them that set-backs are normal in recovery and reminding them to do some self-care to get back on track. In addition to these JITAI components are psychoeducation content available on demand.

Textbox 1. **Content structure of the just-in-time adaptive intervention “Shift.”**

**Onboarding:**

- Welcome and explanation that “Shift” was created by people with lived experience.
- Set up personalized circuit breakers for when anger is out of control.
- Set-up notification schedule for check-ins.
- Switch-off for unwanted information, such as alcohol-related information, menstrual health, and military and first responder.
- Select from one of the 2 coaches, who is a digital voiceover that provides the audio for the app, the welcome message, and the messages provided when an individual reports losing control of their anger.

**Psychoeducation:**

- Anger after trauma.
- Healthy versus unhealthy anger.
- Baseline arousal and how to reduce stress levels using progressive muscle relaxation.
- How anger is linked to military or first responder roles.
- How menstrual health and anger might be connected.
- How diet, exercise, sleep, connection, and substance use is connected to overall well-being.
- Rumination and how to stop it.
- Assertive communication versus unhealthy communication styles.

**Mood monitoring:**

- Four daily check-ins on anger stage (ie, no anger, escalating, out of control, and after a crash; Figure 1).

**Intervention components:**

- When users check-in as having no anger, they are prompted to either practice progressive muscle relaxation, or engage with the psychoeducation components of “Shift*.”*
- When users check-in that their anger is escalating, they are prompted to practice a cognitive-behavioral-therapy–based skill that focuses on the physiological aspect of anger (termed, “body”), the cognitive aspect of anger (termed “mind”), or their behavior when angry (termed “action”). The “body” intervention components are isometric exercises, a targeted form of tension release designed specifically for discharging anger from the body, and cyclic sighing, recognized as the simplest and most effective way to increase relaxation. The “mind” intervention components target stopping anger rumination, and cognitive reframing for anger-provoking situations. The “action” intervention components provide assertive communication skills to use.
- When users check-in that their anger is out of control, they receive one of their circuit breakers established at the onboarding of the app.
- When users check-in that they have had an episode of anger, and behaved in a way they regret, (termed “after a crash”), a warm supportive message and suggestions for self-care are provided by the app coach.

**Crisis:**

- At any time, users can go into the app and receive tailored helpline information to support a crisis state.

## Discussion

### Principal Findings

The aim of this study was to co-design and develop a JITAI for individuals who have experienced trauma and problem anger. We engaged experts by experience and experts by profession to establish design principles and content, as well as opportunities to intervene, resulting in the JITAI “Shift.” Building on previous work that has used mood monitoring for problem anger [2,9], “Shift” is a JITAI that leverages regular check-ins on anger state as the tailoring variables with bite-sized, digitally delivered CBT-based skills to manage lower levels of anger, and a personalized, self-led circuit breaker for when anger is out of control. “Shift” is now developed because of these findings and a micro-randomized trial is being conducted.

From a design perspective, using individual interviews before group workshops was valuable for managing trauma, sensitive personal issues, and the feelings of shame that can accompany problem anger in experts by experience, as it allowed individuals to generate information about the intervention privately, but then also benefit from sharing ideas in a group format. Other methods within the workshop to improve the participant experience include not using digital tools to collect data, which can be frustrating or challenging to learn to navigate, and not getting participants to actively criticize or provide feedback on each other’s ideas. Although there is potential for more work in this area, these findings speak to the importance of research and development of digital mental health tools being co-designed with potential end users. Emerging research is showing that co-design is an important step to ensuring that digital mental health tools are accessible, appropriate, and effective [24]. Without end-user involvement, interventions could miss the mark in terms of developing effective and acceptable digital health programs. Very little research has used co-design in problem anger, or in the design of JITAI. Our findings are consistent with a study that co-designed a JITAI for children to limit sun exposure, in that both opportune times to intervene, and the content and design of the intervention were uncovered as a result of co-design [25].

Several learnings from this study have significant implications for design of digital mental health tools for understudied populations and problem anger more broadly. First, our results showed that individuals with problem anger often try digital mental health tools focused on symptoms of anxiety and depression and can find these anger-provoking. Second, while previous research has established the value of lived experience stories in digital mental health tools for trauma-exposed populations [26], our study found that individuals with problem anger preferred a self-led approach rather than hearing others stories. Third, our results shows that individuals with problem anger have significant unmet needs in the aftermath of a high anger episode. Finally, our results showed that digital mental health tools must balance a focus on changing pathological thoughts, feelings, and behavior with providing warmth, empathy, validation, and support, all of which are typically provided via the therapeutic relationship in psychological treatment and are at risk of being lost in digital approaches. Important next steps are evaluating the components of “Shift” using methods such as a micro-randomized trial, followed by a randomized controlled trial to validate efficacy.

### Limitations

While the study had diversity in terms of gender, some representation of disability, and a diversity of trauma experiences, this study is limited by a sample that contained English-speaking individuals only. In addition, the study only collected qualitative data and work is needed to pilot the intervention to determine acceptability and feasibility of the intervention to individuals who have experienced trauma and have problem anger. Finally, EMA-driven JITAIs can become burdensome for participants, and innovations in sensor-based detection of mood states will likely advance “Shift” [27]. As yet, preliminary work in detecting anger in this population from wearables has found promising results that remain technically challenging to deploy, and sensor-based JITAIs in this space warrant further investigation [28].

## Supplementary material

10.2196/62960 Multimedia Appendix 1 Interviews.

## Abbreviations

CBT: cognitive behavioral therapy
JITAI: just-in-time adaptive intervention
NIMH: National Institute of Mental Health
TRIRP: Trauma and Resilience Informed Research Principles and Practice
