# Supplementary material for: A Just-in-Time Adaptive Intervention (Shift) to Manage Problem Anger After Trauma: Co-Design and Development Study
Source: JMIR Hum Factors. 2025 May 22;12:e62960. doi: 10.2196/62960 (PMC12148248; doi:10.2196/62960)
Supplement: Multimedia Appendix 1 [file humanfactors-v12-e62960-s001.docx]

**Interviews**

**Interviewer instructions:** The aim of this interview is to gain unique information from the participant about his/her experience and views, so it is important to use open-ended, exploratory questions that invite the participant to provide a perspective.

**Trauma-informed research**

**Interviewer instructions:** Participants in this study have experienced trauma. By it’s very nature, the research process has potential to replicate power dynamics and invalidation that are core to many trauma experiences. At all times, conduct this interview with principles of safety, empowerment, and control in the hands of the participant:

1. Check participants know upfront what will be discussed in the study and why
2. Remind participants they do not have to discuss anything they don’t want to
3. Use strengths-based respectful language
4. Check participants know they can end the interview at any time without repercussions
5. Monitor distress throughout

**Introduction**

Thank you for agreeing to meet with us today. (Introduce interviewers).

We’re looking forward to hearing your thoughts about a new smartphone intervention we are developing with your help. These interviews typically last about an hour. Will that work for you today? First, we'll take the opportunity to talk about your digital usage, and then talk about what you’d like in terms of content, features, and functionality.

**Digital usage**

- Can you tell us about any mobile apps you have tried for health, self-help, mood, wellbeing? What did and didn’t you like?
- Have you used relaxation or mindfulness apps? Did you find them helpful? (Prompt if needed as to whether they found it helpful for anger or other mood states).
- If you were to use an app to manage your anger, in what context would you use this app? Where/how it would fit into your daily life?
- Thinking about your anger intensity on a scale of one to ten, with 10 being your most intense anger, how would you want to use a smartphone app to manage your anger if your anger was under 4? What about if your anger was over 7?
- Is there anything about your data plan, or phone that would impact you being able to access content?

.

**Content**

- Thinking of any of your past experiences in self-managing your anger, what has been helpful? What hasn’t been?
- Thinking about the content of the app, how helpful would it be if the app contained content on:
  - explaining the causes of anger after trauma?
  - support around potential triggers of anger, like poor sleep, or parenting stress?
  - external connections to other supports?
  - ways of managing aggression?
- How would you like to have content delivered to you? Sometimes in apps we deliver information from experts. Would you want to hear from experts? If so, what type of experts? Would you want to hear from other people who have recovered?
- Sometimes people find that mental health apps that also focus on strengths can support engagement. Is there anything you can think of that might draw on your strengths that we can use in the app, as opposed to just focusing on areas of improvement?
- Sometimes people find that motivations to use the app, sometimes called gamification, which can look like winning points or progressing through a mini game can help engagement. Is that something that you would like?

**Look/feel**

- Thinking about the language around mental health, are there terms relating to anger, stress, and triggers that you prefer or dislike?
- It we wanted to feedback information to you via an app on your anger/mood what is a meaningful and engaging way for us to do this?
  - Prompts: Visually? Over a week/day? Patterns?
- Do you have any specific look and feel preferences for a mental health/mental wealth/wellbeing app that was focused on managing anger?
- Thinking of preferred features, would you want videos, activities, audio/coach support?
- Can you tell us what the minimum and maximum contact per day would be, in terms of notifications and prompts to engage with the app?

**Wearable**

- Do you have your own personal smartwatch or wearable? Do you use it to track your mood or stress levels?

**Conclusion**

I really value you taking the time to share that with us. Sometimes it’s not until a day or two later that you may think of something else, you are welcome to follow-up if that’s the case.
